# Supplementary material for: The IgCAM CAR Regulates Gap Junction-Mediated Coupling on Embryonic Cardiomyocytes and Affects Their Beating Frequency
Source: Life (Basel). 2022 Dec 21;13(1):14. doi: 10.3390/life13010014 (PMC9866089; doi:10.3390/life13010014)
Supplement: Supplementary file 1 [file life-13-00014-s001.zip › life-2040577-supplementary.pdf]

Supplemental Figures and information

Figure S1

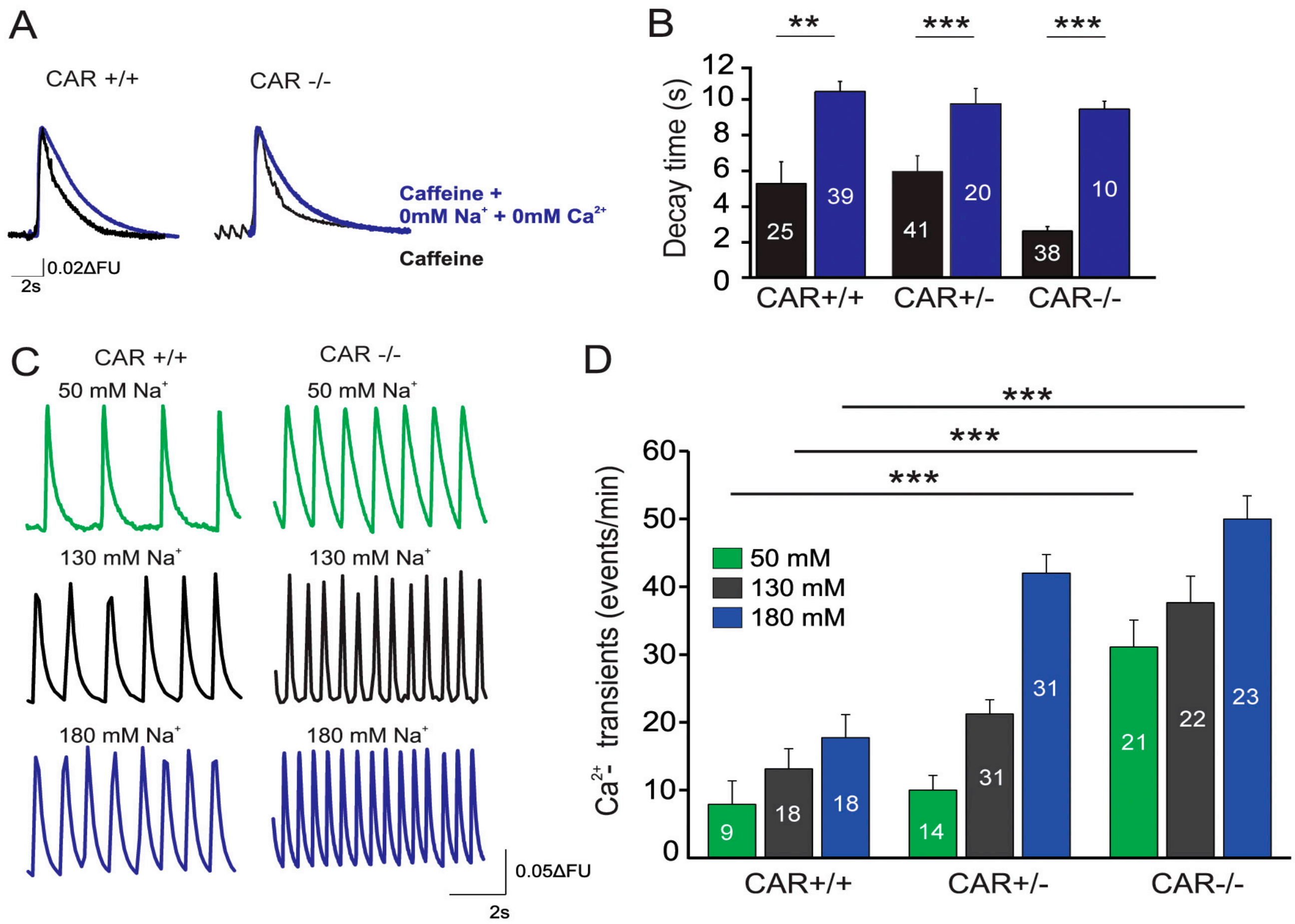

Contribution of NCX to calcium extrusion in CAR wild-type and knockout.

(A,B) To study the role of NCX further decay times of caffeine-induced calcium transients were analysed in ACSF without sodium and calcium. Under these experimental conditions NCX is completely blocked. Consequently, decay times of calcium transients increased dramatically for all genotypes. However, no differences between wild type and knockout were observed supporting our conclusion that NCX contributes to the increased extrusion of calcium in CAR knockout. (C,D) The activity of NCX can be manipulated by variations of the driving force, e.g. by changes of the extracellular sodium concentration. Decreasing extracellular sodium concentration from 130 mM to 50 mM caused a strong decrease in the spontaneous beating frequency while an increase in NCX activity by raising the extracellular sodium concentration from 130 mM to 180 mM resulted in increased beating of cardiomyocytes of all genotypes. However, comparison of wild type and knockout always revealed a stronger increase in knockout cardiomyocytes also suggesting a stronger induction of NCX activity in CAR knockout cardiomyocytes. ** $p < 0.01$, *** $p < 0.001$.

Figure S2

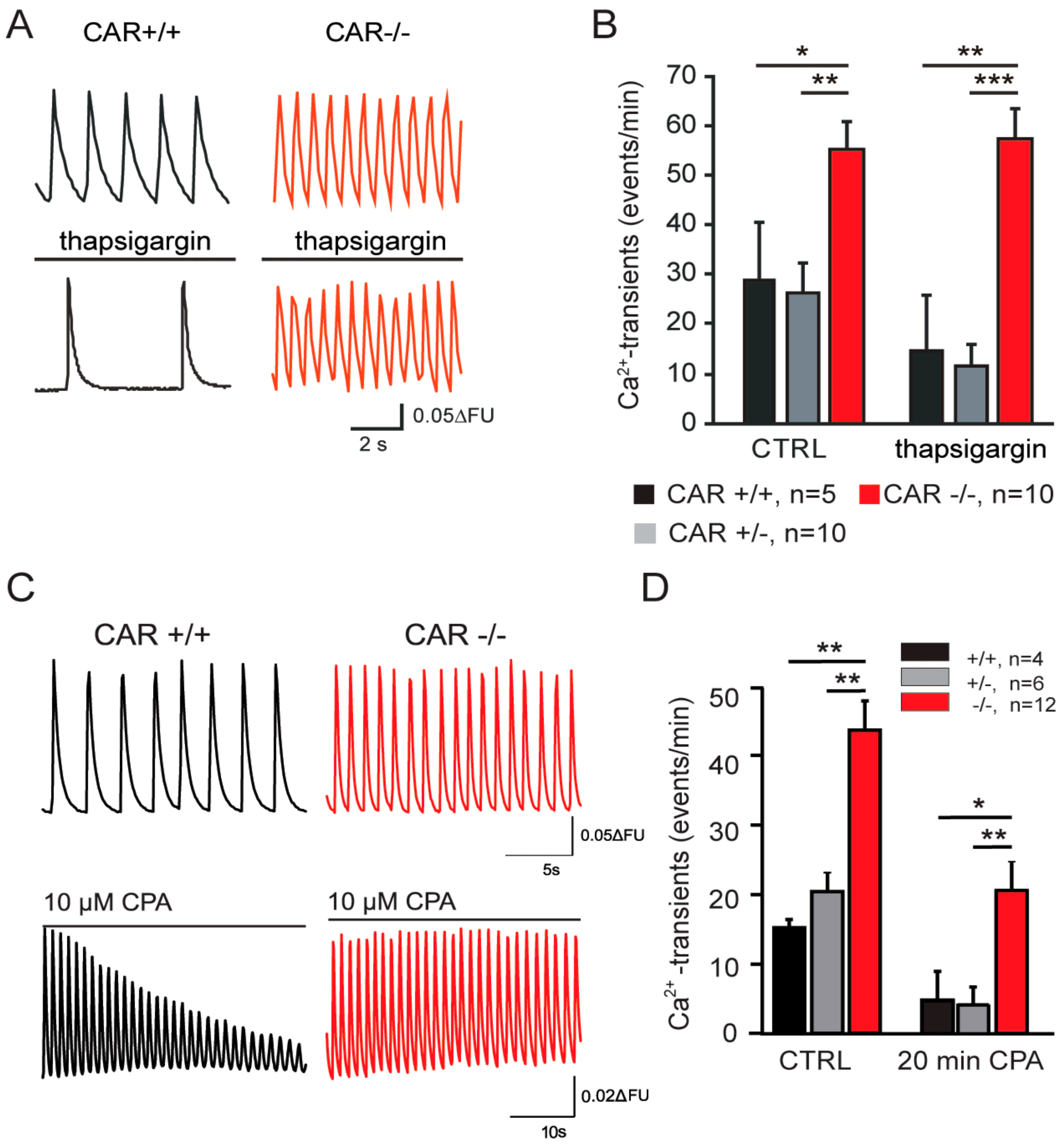

Thapsigargin or CPA only partially reduced the activity of SERCA2 in the absence of CAR.

(A,B) Recording of calcium transients during a 20 minutes period showed a strong reduction of the beating frequency of wild type cardiomyocytes after application of 10 μM thapsigargin. In contrast, CAR knockout cardiomyocytes did not change their spontaneous beating frequency indicating that SERCA2 either becomes less sensitive to thapsigargin. In (A) individual traces are shown. (C,D) Application of the SERCA2 inhibitor CPA at 10 μM only partially reduced the

beating frequency within 20 minutes recording period in CAR-deficient in contrast to wild type cardiomyocytes. In wild type cardiomyocytes amplitudes were already strongly reduced after 1 minute of application of CPA. In C) individual traces and in (D) a summary of measurements is shown. * $p < 0.05$, ** $p < 0.01$, *** $p < 0.001$.

Figure S3

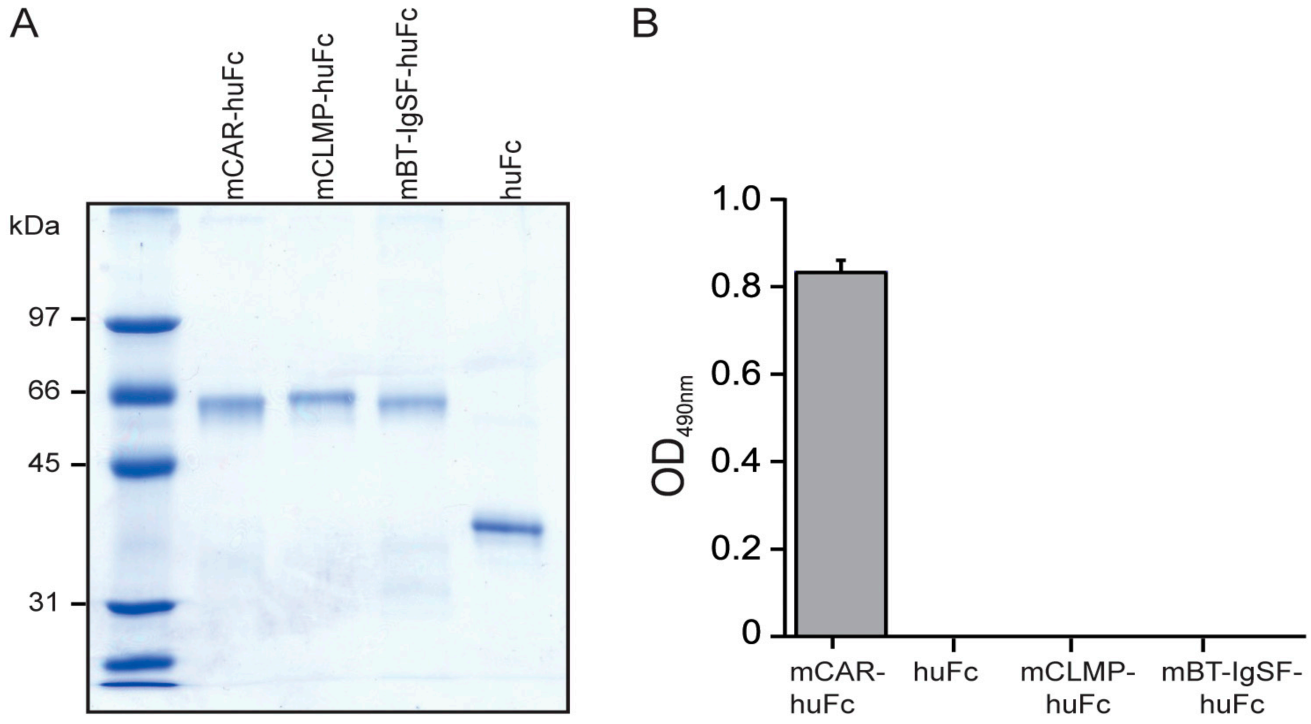

The fiber knob Ad2 binds to the extracellular segment of CAR but not to CLMP or BT-IgSF.

(A) SDS-PAGE of purified Fc fusion proteins analysed by SDS-PAGE. (B) Fc fusion proteins of mCAR, mBT-IgSF, mCLMP or the Fc-fragment at a concentration of 1 µg/ml were immobilised on ELISA plates (200 µl) followed by blocking of residual binding sites and by incubation with 200 µl fiber knob Ad2 at a concentration of 125 ng/ml. Binding was detected by an HRP-conjugated monoclonal antibody directed to the His-tag of Ad2. Binding of Ad2 was only observed to mCAR-Fc but not to BT-IgSF-Fc, CLMP-Fc or to the Fc-fragment (n=3).

Supplemental Figure S4

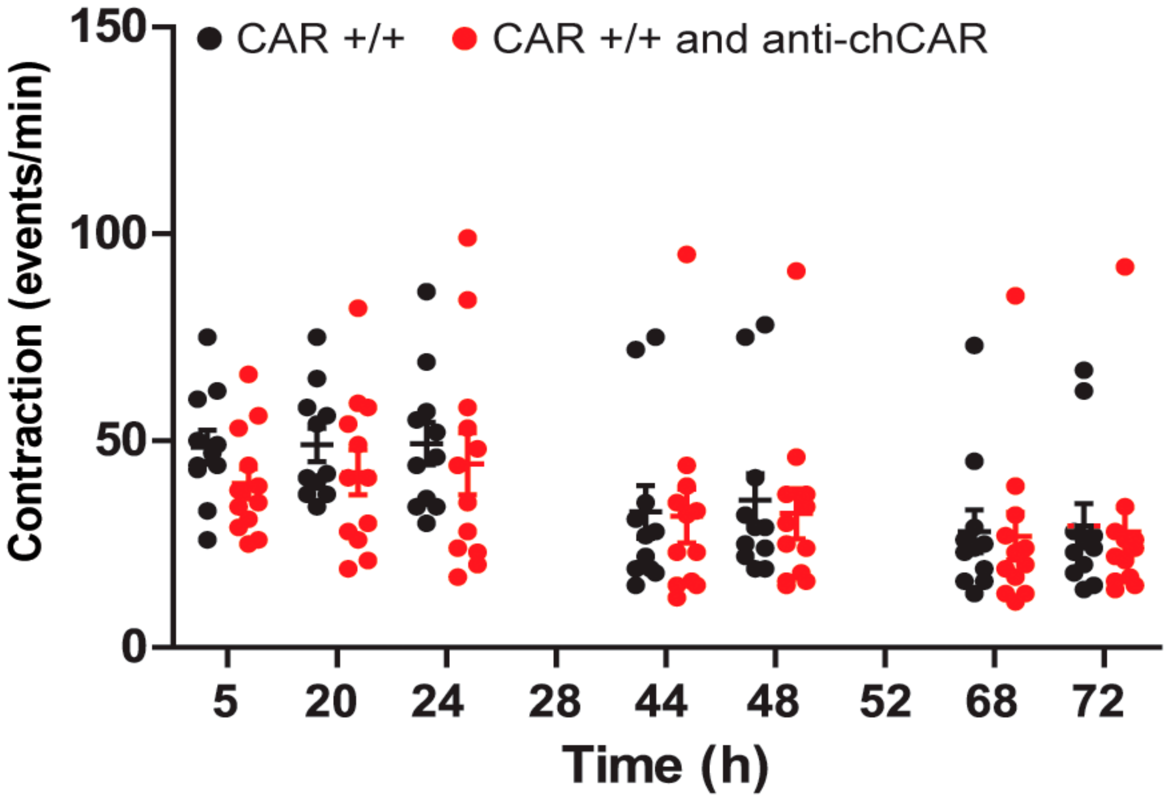

Analysis of spontaneous heart beating in the presence of rabbit antibodies to chick CAR.

Wild type cardiomyocytes were cultured in the presence of the IgG fraction of rabbit antibodies to chick CAR (Rb54, at 0.5 mg/ml) and the spontaneous beating was counted. Rabbit antibodies to chick CAR do not recognize mouse CAR [2]. 12 wild type hearts treated with Rb54 and 11 hearts as controls were evaluated ($p=0.199$; two-way ANOVA).

Supplemental information: Quantification of calcium extrusion rate constants

The rate constants k (k = 1/decay time constant) of SERCA2, NCX, PMCA and mitochondria were calculated according to Voigt et al. (2012). The systolic calcium decline is the combined extrusion of calcium by SERCA2, NCX, PMCA and mitochondria. Therefore, the following Equation (S1) can be defined:

$$k_{syst} = k_{SERCA2} + k_{NCX} + k_{PMCA} + k_{mitochondria} \quad \text{(S1)}$$

Application of caffeine triggers a complete release of calcium from SR and uptake of cytosolic calcium by SERCA2 is much slower compared to the fast release of calcium. Therefore, during analysis of the calcium decline of caffeine triggered calcium transients SERCA2 is disregarded. The rate constant of caffeine induced calcium transients consequently is composed of the NCX, PMCA and mitochondria extrusion:

$$k_{Caff} = k_{NCX} + k_{PMCA} + k_{mitochondria} \quad \text{(S2)}$$

$$k_{SERCA2} = k_{syst} - k_{Caff} \quad \text{(S3)}$$

To determine the NCX rate constant Na^+- and calcium-free ACSF was applied which inhibits NCX activity completely. The cardiomyocytes were treated with 10 mM caffeine and the calcium decline was analyzed . Due to the lack of SERCA2 and NCX activity the calcium extrusion is only performed by PMCA and mitochondria and the following equation (S4) can be defined and the rate constant of NCX can be calculated (S5):

$$k_{0Na+0Ca} = k_{PMCA} + k_{mitochondria} \quad \text{(S4)}$$

$$k_{NCX} = k_{Caff} - k_{0Na+0Ca} \quad \text{(S5)}$$

The influence of PMCA on calcium extrusion was determined by blocking NCX activity and mitochondria calcium uptake with 20 μM Ru360. The caffeine induced cytosolic calcium increase is only reduced by PMCA. Therefore, the rate constant of PMCA can be determined by equation (S6) and consequently the rate constant of mitochondria by (S7):

$$k_{0Na+0Ca,Ru360} = k_{PMCA} \quad \text{(S6)}$$

$$k_{mitochondria} = k_{0Na+0Ca} - k_{PMCA} \quad \text{(S7)}$$

Supplemental Table S1: Affymetrix gene microarray analysis (provided as Excel sheet, see also data base entry GEO GSE138831)

Supplemental Table S2: Primer sequences for real-time PCR

To confirm microarray data the expression of several genes was analysed by quantitative RT-PCR and normalized to actin. The data supported the results obtained by Affymetrix microarrays and did not show significant changes of the level of mRNA between wild type and CAR-deficient embryonic hearts for the genes listed below (data not shown).

Gene	Primer for	Primer rev	Product (bp)	p-value	n
Serca2a	CTGTGGAGACCCTTGGTTGT	CAGAGCACAGATGGTGGCTA	245	0.118	7
PLB	AGCTGGGACCAAAGGAACTT	TAGCCGAGCGAGTGAGGTAT	223	0.257	4
Ncx1	AGCTCTCCTGGAGTTGTGGA	TGGAAGCTGGTCTGTCTCCT	184	0.316	7
RyR2	TGCCGAGGTCTTCTCAAAGT	TGCTTTAGTCGTGAGGGCTT	228	0.259	13
IP3R2	ATGGCGAGGGTCTGTAACAC	GCAGCTGGATAAAGACTGGC	166	0.938	13
Cx40	CTCTAAACGTGGAAGGCTCG	TGAACAGGACAGTGAGCCAG	136	0.174	12
Cx43	GAACACGGCAAGGTGAAGAT	GACGTGAGAGGAAGCAGTCC	187	0.183	12

Supplemental Table S3: Antibodies for immunohistochemistry or immunocytochemistry and for Western blotting

Antibody	IHC and ICC	Western blots	Source
Rabbit anti-mCAR-Fc (Rb80) IgG	1-3 µg/ml	1 µg/ml	Patzke et al., 2010
Rabbit anti-chCAR-Fc (Rb54) IgG		1 µg/ml	Patzke et al., 2010
Rabbit anti-mL1-CAM		1 µg/ml	Rathjen and Schachner, 1984
mAb anti-Cx43	1 µg/ml		Transduction Laboratories, #610061
Rabbit anti-Cx43	1:200	1:1000	Cell signaling, #3512
mAb anti-Cx40 (B-3)		1 µg/ml	Santa Cruz #sc-365107
mAb anti-Cx45 (G-7)		1 µg/ml	Santa Cruz #sc-374354
mAb anti-Cx50		1 µg/ml	Invitrogen #33-4300

mAb anti-β-Catenin		0.5 μg/ml	Transduction Laboratories #610153
mAb anti-ZO-1	5 μg/ml		Invitrogen #339100
Rabbit anti-ZO-1	1:100		Invitrogen #40-2200
mAb anti-sacomeric α-actinin (EA-53)	1:800		Sigma-Aldrich, #A-7811
mAb anti-GAPDH (1D4)		0.5 μg/ml	Novus Biologicals, #NB300-221
mAb anti-Clathrin (heavy chain)		1:1000	BD Transduction Laboratories #610499
Rabbit anti-phospholamban		1:1000	Cell Signaling #8495
mAb anti-Penta-His		1:2000	Qiagen #34660
Goat anti-Rabbit-Cy3	1:400 – 1:1000		Dianova
Goat anti-Mouse-Alexa488	1:400 – 1:1000		Molecular Probes
Goat anti-Rabbit-HRP		1:20 000	Dianova
Goat anti-Mouse-HRP		1:20 000	Dianova
Rabbit anti-Akt		1:1000	Cell Signaling #9272
Rabbit anti-pAkt		1:1000	Cell Signaling #9271
